# Supplementary material for: Quantification of Volatile Aldehydes Deriving from In Vitro Lipid Peroxidation in the Breath of Ventilated Patients
Source: Molecules. 2021 May 21;26(11):3089. doi: 10.3390/molecules26113089 (PMC8196825; doi:10.3390/molecules26113089)
Supplement: Supplementary file 1 [file molecules-26-03089-s001.zip › molecules-1205627-supplementary/Supplementary File 1 - Calibration.pdf]

# *Supplementary File 1 – Calibration:*

# Quantification of volatile aldehydes deriving from in vitro lipid peroxidation in the breath of ventilated patients

Lukas M. Müller-Wirtz MD[1,2], Daniel Kiefer MD[1], Sven Ruffing Cand. Med.[1], Timo Brausch MD[1], Tobias Hüppe MD[1,2], Daniel I. Sessler MD[2,3], Thomas Volk MD[1,2], Tobias Fink MD[1,2], Sascha Kreuer MD[1,2], Felix Maurer DSc[1,2]

1) **CBR** - **C**enter of **B**reath **R**esearch, Department of Anaesthesiology, Intensive Care and Pain Therapy, Saarland University Medical Center, Homburg (Saar), Germany

2) **O**UTCOMES **R**ESEARCH Consortium, Cleveland, Ohio, USA.

3) Department of **O**UTCOMES **R**ESEARCH, Anesthesiology Institute, Cleveland Clinic, Cleveland, OH, USA

**Corresponding Author:**

Lukas Martin Müller-Wirtz, MD
CBR - Center of Breath Research
Department of Anaesthesiology, Intensive Care and Pain Therapy
Saarland University Medical Center and Saarland University Faculty of Medicine
66421 Homburg / Saar, Germany
Phone: +4968411622758
Email: lukas.wirtz@uks.eu
ORCID: 0000-0002-7984-1798

## Experimental setup

A perfluoroalkoxy alkane calibration flask was used with three ports for tubing and one port for a fan (BOLA, Grünsfeld, Germany). The exact volume of the flask was measured by filling it with water. The flask was placed into a water bath (Precision GP10, Thermo Scientific, Waltham, USA) and heated to 37° C (310.15 K). An overhead stirrer (Heidolph, Schwabach, Germany) was used to drive a fan inside the flask. Ports were connected with perfluoroalkoxy alkane tubes to an MCC-IMS (BreathDiscovery, B&S Analytik, Dortmund, Germany), active charcoal filtered nitrogen (NM32LA, Peak Scientific, Inchinnan, UK), and synthetic air (Alphagaz 1, Air liquide, Düsseldorf, Germany). The flask was cleaned by flushing with nitrogen for several hours prior to calibration (Figure S1).

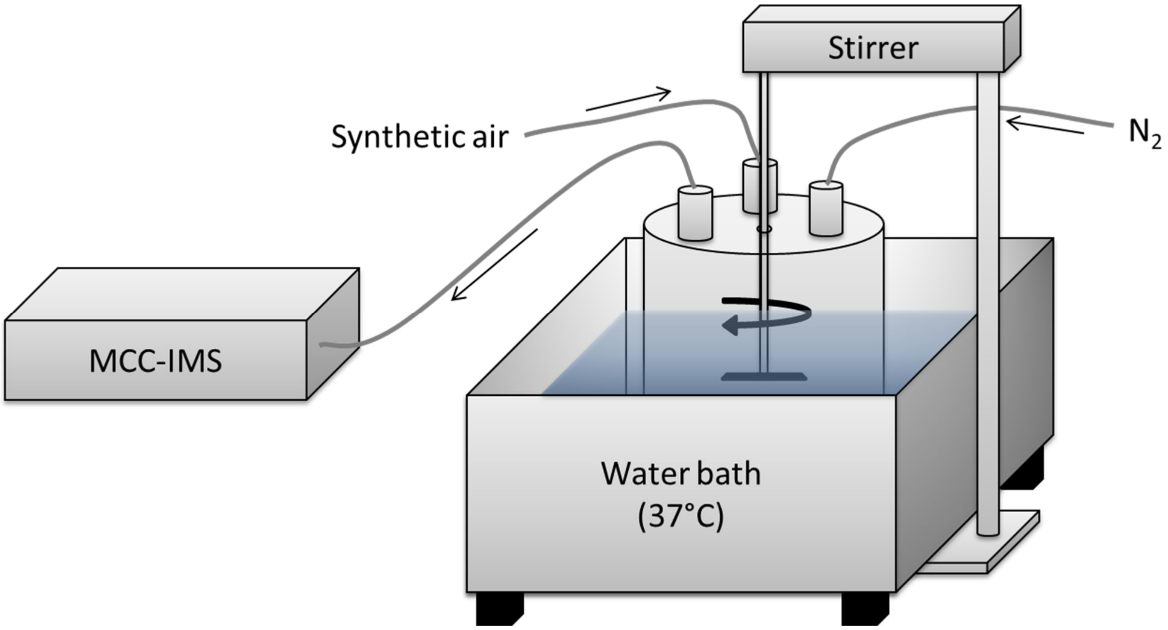


**Figure S1:** Calibration setup

Calibration procedure: 1. Flushing with $N_2$ and/or synthetic air, 2. Blank sample, 3. Injection of hexane-diluted standard, 4. Stirring (2 min), 5. Sample (MCC-IMS measurement).

## Stock solution and calibration procedure

The reference substance was evaporated in several defined concentrations in the flask and measured with the IMS to create a calibration curve. Eight different concentrations were produced for each calibration by diluting the reference substance with n-hexane (Pestinorm Supra Trace, VWR, Darmstadt, Germany). After evaporation, 1 µL corresponded to 0.156, 0.312, 0.625, 1.25, 2.50, 5.00, 7.50, 10.0, and 20.0 ppb inside the flask (octanal: 0.1 – 12.5 ppb, decanal: 0.2 – 100 ppb). The required amounts of reference substances for the highest standard were calculated from the volume of the flask using the ideal gas equation (Supplementary File 2 – Calibration Calculation Sheet). Lower concentrated standards were made by geometrical dilution of the highest standard with n-hexane.

**Sample calculation for preparing a stock solution for pentanal calibration**

**(with 1 µL generating 20 ppb in the flask)**

1. 1 µL of pentanal (98%) has a gaseous volume of 0.23 mL (purity: 98%, density: 810 µg/µL, molar mass: 86.13 g/mol, temperature: 310.15 K, pressure: 101325 Pa)
2. 1 µL produces a concentration of 87580 ppb in the calibration flask with a volume of 2678 mL
3. 20 µL pentanal is diluted with 87.56 mL hexane as a stock solution

Calibration was performed from lowest to highest concentration. Each concentration was generated and measured three times to assess reproducibility (standards of octanal and decanal were measured once, as both aldehydes were neither detected from oxidizing polyunsaturated fatty acids nor in breath). Flushing of the calibration flask with nitrogen or synthetic air was followed by assessment of a blank sample to confirm that the flask was indeed clean. 1 µL of the respective reference dilution was then pipetted into the flask, including the pipette tip with subsequent fan mixing for two minutes and sampling.

The calibration curves for the volatile aldehydes that were quantified in the headspace of oxidizing polyunsaturated fatty acids are presented in figure S2. The mean relative standard deviation for the calibrations of pentanal, hexanal, heptanal, and nonanal was 5.8% (min. 0.3% – max. 17%). Pentanal calibration gave three measurements with a relative SD above 20%, which was corrected by the exclusion of outliers.

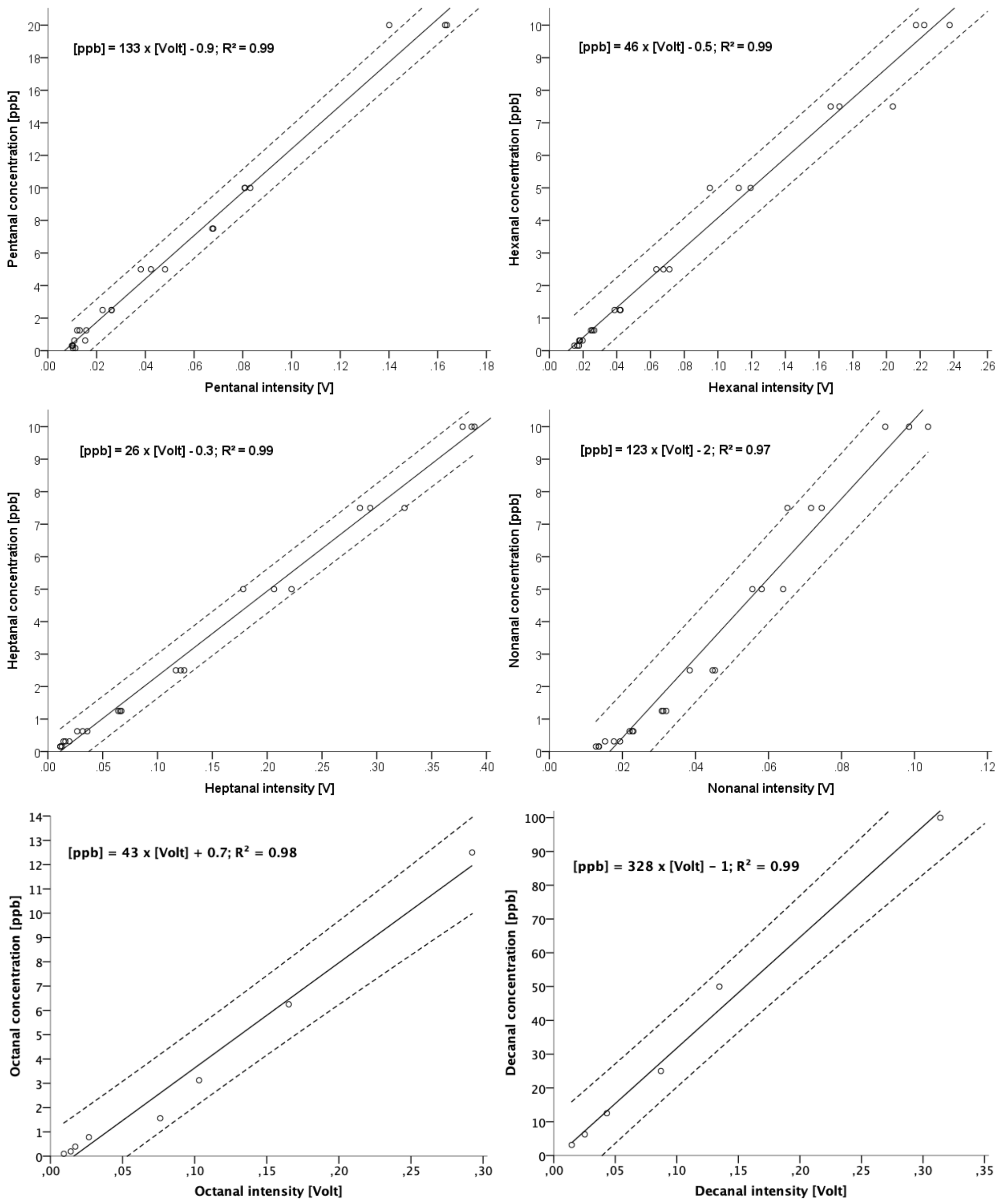


**Figure S2:** Calibration curves
